# A novel approach for obtaining a near complete dataset for time-to-event outcomes in the bronchiolitis of infancy discharge study (BIDS) using an informed multiple imputation technique

**DOI:** 10.1186/1745-6215-16-S2-P146

**Published:** 2015-11-16

**Authors:** Aryelly Rodriguez, Steff Lewis

**Affiliations:** 1 University of Edinburgh, Edinburgh, UK

## 

Time to cough resolution was the primary outcome for the BIDS trial (http://www.nets.nihr.ac.uk/projects/hta/099116). Patients were followed up at 7, 14, 28 days and 6 months after randomisation. Strenuous efforts were made by the research team in order to achieve a near complete data set. However there were still some parents/guardians who could not recall the date on which their infant stopped coughing, but who could recall whether their infant had stopped coughing since last followed up.

We assumed that the lost event dates were missing at random. So we developed a multiple imputation technique: If it was known that the event occurred, but the precise date was unknown, a value was chosen between the date that the event was last known to not be present and the date of the follow up when it was present. Then a value was chosen at random from patients in the same treatment group whose cough stopped in a similar time frame. This process was repeated 100 times, and the analysis done on each dataset. The mean values for the estimate of the median and its CI limits were used for reporting.

In this way we managed to increase the number of available event dates from 507 (82% of 615) to 589 (96% of 615). By introducing simple “yes/no event occurred” questions at each follow-up visit and the usage of programmatic tools we recovered around 13.4% of the data with the informed imputation technique. The imputed and complete case analyses reached the same conclusion.

